# Public preferences for using quantitative faecal immunochemical test versus colonoscopy as diagnostic test for colorectal cancer: evidence from an online survey

**DOI:** 10.3399/bjgpopen20X101007

**Published:** 2020-02-05

**Authors:** Christian von Wagner, Wouter Verstraete, Yasemin Hirst, Brian D Nicholson, Sandro T Stoffel, Helga Laszlo

**Affiliations:** 1 Reader, Research Department of Behavioural Science and Health, University College London, London, UK; 2 Research Assistant Research Department of Behavioural Science and Health, University College London, London, UK; 3 Senior Research Fellow, Research Department of Behavioural Science and Health, University College London, London, UK; 4 Senior Clinical Researcher, Nuffield Dept Primary Care Health Sciences, University of Oxford, Oxford, UK; 5 Research Associate, Research Department of Behavioural Science and Health, University College London, London, UK; 6 European Center of Pharmaceutical Medicine, University of Basel, Basel, Switzerland; 7 Programme Manager, UCLH Cancer Collaborative, University College London Hospitals, London, UK

**Keywords:** diagnostic tests, preference elicitation, choice experiment, colorectal neoplasms, primary health care, surveys and questionnaires

## Abstract

**Background:**

There has been interest in using the non-invasive, home-based quantitative faecal immunochemical test (FIT) to rule out colorectal cancer (CRC) in high-risk symptomatic patients.

**Aim:**

To elicit public preferences for FIT versus colonoscopy (CC) and its delivery in primary care.

**Design & setting:**

A cross-sectional online survey in England.

**Method:**

A total of 1057 adults (without CRC symptoms and diagnosis) aged 40–59 years were invited from an English online survey panel. Responders were asked to imagine they had been experiencing CRC symptoms that would qualify them for a diagnostic test. Participants were presented with choices between CC and FIT in ascending order of number of CRCs missed by FIT (from 1–10%). It was measured at what number of missed CRCs responders preferred CC over FIT.

**Results:**

While 150 participants did not want either of the tests when both missed 1% CRCs, the majority (*n* = 741, 70.0%) preferred FIT to CC at that level of accuracy. However, this preference reduced to 427 (40.4%) when FIT missed one additional cancer. Women were more likely to tolerate missing CRC when using FIT. Having lower numeracy and perceiving a higher level of risk meant participants were less likely to tolerate a false negative test. Most of those who chose FIT preferred to return it by mail (62.2%), to be informed about normal test results by letter (42.1%), and about abnormal test results face to face (32.5%).

**Conclusion:**

While the majority of participants preferred FIT over CC when both tests had the same sensitivity, tolerance for missed CRCs was low.

## How this fits in

Hypothetical vignette studies have demonstrated a strong preference for CRC testing in low-risk scenarios. This was the first study to investigate users’ attitudes towards different CRC tests to rule out significant colorectal diseases when they experience symptoms.

This study showed that while most people with symptoms would prefer FIT over CC if both tests miss 1% of CRCs, a large proportion of participants switched their preference to CC as soon as FIT missed more CRCs than CC. A smaller but important subgroup of people exhibited a strong preference for non-invasive testing (that is, using FIT over CC) even if the test missed 10% of CRCs. Preferences for FIT rollout were not universal but varied by demographic background, and test result.

## Introduction

CRC is the fourth most common cancer in the UK and the second biggest cause of cancer deaths.^1^ The majority of CRCs are still diagnosed among symptomatic patients when outcomes tend to be worse in terms of survival.^2,3^ There is strong impetus to promote early symptomatic presentation and prompt referral. One of the biggest barriers to prompt diagnosis is that symptoms of CRC are common and non-specific. CRC symptoms regarded as high-risk, such as rectal bleeding, only have a 3–4% positive predictive value.

In England, patients with symptoms suggestive of CRC are referred urgently via the 2-week wait (2WW) pathway (see Box 1 for a more detailed description of qualifying symptoms for different age groups).^4,5^ The majority of these patients complete a CC, which is the gold standard test to rule out CRC, but the procedure carries risks and opportunity costs for patients, as well as costs to the health service. As a result, there has been interest in using a non-invasive FIT to rule out CRC in patients with symptoms qualifying for 2WW referral in a much safer (avoiding the 0.03–0.7% risk of bowel perforation with CC) and more cost-effective way (the average price of colonoscopy is £372 compared with £5 per FIT kit).^6,7^

Box 1 **Criteria for 2-week-wait referral**

**National Institute for Health and Care Excellence (NICE)guidance** ^4^

1.3.1 Refer adults using a suspected cancer pathway referral (for an appointment within 2 weeks) for colorectal cancer if:

- they are aged ≥40 years with unexplained weight loss and abdominal pain **or**
- they are aged ≥50 years with unexplained rectal bleeding **or**
- they are aged ≥60 years with:
  - iron‑deficiency anaemia **or**
  - changes in their bowel habit, **or**
- tests show occult blood in their faeces **(new 2015)**

1.3.2 Consider a suspected cancer pathway referral (for an appointment within 2 weeks) for colorectal cancer in adults with a rectal or abdominal mass **(new 2015)**

****

1.3.3 Consider a suspected cancer pathway referral (for an appointment within 2 weeks) for colorectal cancer in adults aged <50 years with rectal bleeding **and** any of the following unexplained symptoms or findings:

- abdominal pain
- change in bowel habit
- weight loss
- iron‑deficiency anaemia **(new 2015)**

1.3.4 This recommendation has been replaced by diagnostics guidance on quantitative faecal immunochemical tests to guide referral for colorectal cancer in primary care. The diagnostics guidance recommends tests for occult blood in faeces, for people without rectal bleeding but with unexplained symptoms that do not meet the criteria for a suspected cancer pathway referral inrecommendations 1.3.1 to 1.3.3.

Previous studies have reported that a negative FIT test could rule out CRC, with a negative predictive value of 96–100%, and free-up CC capacity, but a small proportion of patients with bowel cancer could receive false negative results, which could raise concerns.^8–11^ This is particularly relevant when the prevalence of disease is low, such as in patients attending primary care. A recent survey indicated that just over one-third of GPs preferred to use a FIT as a rule-out test over 2WW referral, but showed confusion over the optimal use of FIT in symptomatic patients.^12^ GPs who were relatively more likely to make urgent referrals (more than 10 referrals per year) were less willing to use FIT. Conversely, believing that FIT was highly accurate was the strongest positive predictor of wanting to use FIT as a triage test.

In addition to considering acceptability among relevant healthcare providers, it is also important to understand the attitudes of patients towards completing the test: only if users find the FIT process acceptable are they likely to complete it satisfactorily and follow the associated investigative pathway.^13,14^ Previous research suggests that asymptomatic participants in the bowel cancer screening programme share GPs' concerns about the accuracy of FIT to detect CRC.^15–17^ The only study in the symptomatic context used a hypothetical vignette study to investigate preferences for diagnostic testing for cancer (including CRC). Participants expressed a clear preference for diagnostic testing at all risk levels (including a risk as low as 1%).^18^ However, no research has been carried out investigating users’ attitudes towards different CRC tests to rule out significant colorectal diseases when they experience symptoms.

The present study aimed to test how public preferences varies between two diagnostics tests (FIT versus CC) at different missed cancer rates using a series of hypothetical choice scenarios. Additionally, the study wanted to elicit preferences for FIT-specific test procedures, such as mode of returning the test kit and preferred way of communicating test results.

## Method

**Figure 1.**
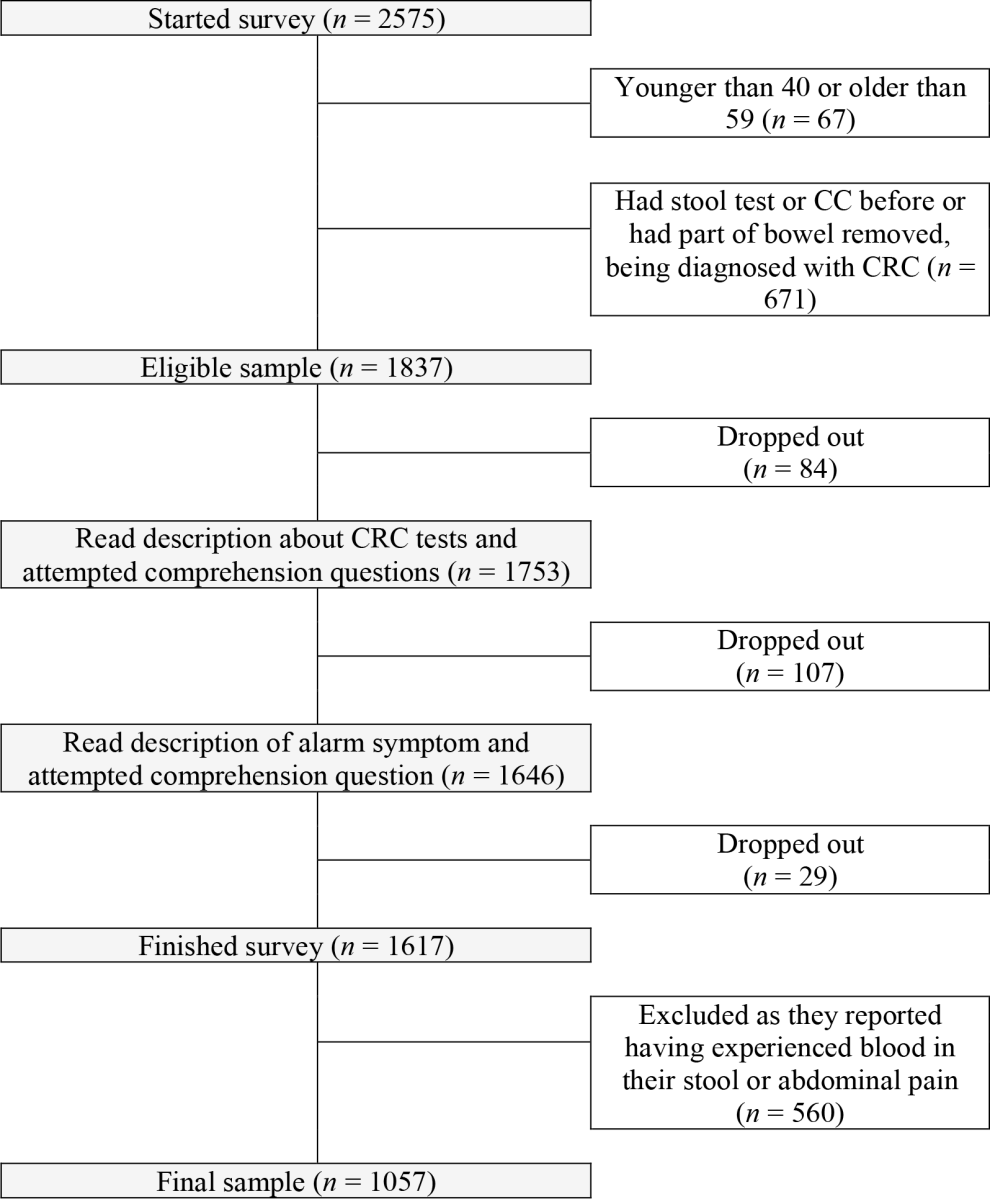
Flow through the survey

A total of 2508 men and women aged 40–59 years living in England were invited to participate from an English online survey panel (Survey Sampling International, now known as Dynata) in November 2018. Figure 1 outlines the various points at which people were excluded from or dropped out of the study. In total, 671 (26.8%) were excluded because they had previously completed a CC or stool test and had been diagnosed with CRC or had parts of their bowel removed.

Those who were eligible to become participants (*n* = 1837) were provided with a description of CC and FIT and were asked to imagine they had been experiencing CRC symptoms (40–49-year-olds: unexplained weight loss and abdominal pain; 50–59-year-olds: unexplained rectal bleeding) that would qualify them for 2WW referral as per the National Institute for Health and Care Excellence (NICE) guideline NG12 (Box 1).^4^

Before participants completed the survey, they were asked to answer a few pre-screening questions in order to ensure that they understood the survey instructions. Those who could not correctly recall their communicated symptom(s) or did not correctly answer four comprehension questions about CC and FIT were asked to repeat the question until they had chosen the correct answer (see questions in the online depository: https://osf.io/ta4bc/).

**Figure 2.**
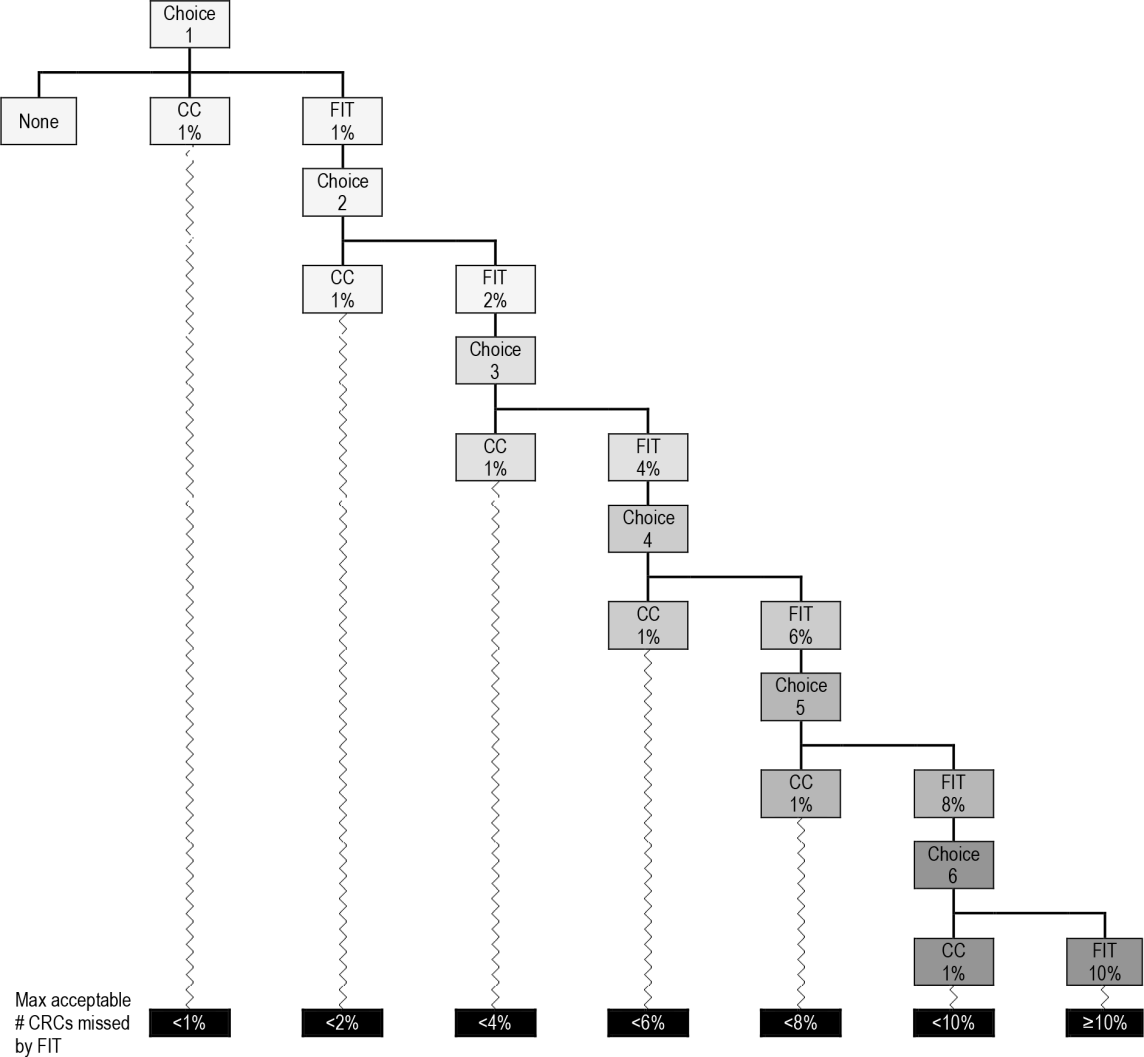
Dynamic forced-choice staircases CC = colonoscopy. CRC = colorectal cancer. FIT = faecal immunochemical test. Max = maximum.

Once people successfully recalled the hypothetical symptoms, participants were asked to choose between the two tests to investigate their hypothetical symptoms. Specifically, a dynamic forced-choice staircase method was used to present participants with a series of binary comparisons between CC and FIT (see Figure 2).^19,20^ The staircase method has been extensively used in psychophysics^19^ to establish a threshold level. Its main advantage is its efficiency as it requires the presentation of many fewer stimuli than other traditional methods; in this case, a discrete choice experiment was used because it enabled the authors to focus on varying a single attribute (namely cancer miss rate), as previous evidence had identified this as the primary concern in patients and healthcare providers.^12,15–17^

CC was reported to miss 1% of CRCs in all scenarios while the CRCs missed by FIT were reported in ascending order ranging from 1–10%. Those who either chose CC in the first scenario or stated that they did not want either test were asked to indicate the rationale for their choice by choosing from a list of reasons. Furthermore*,* participants who preferred CC by default were asked what else they would do about their hypothetical symptom(s). Possible answer options included going back to the GP; monitoring or managing symptom(s); avoiding thinking about the symptom(s); nothing; and something else. For all remaining responders, the point at which they switched from choosing FIT to CC provided the maximum acceptable proportion of missed CRCs (or the minimum acceptable sensitivity for FIT).

### Preferences of FIT procedure

Participants who chose FIT in at least one comparison were asked several questions about the procedure. These included whether they would prefer to return the FIT in person to their GP or by freepost; how and by whom they would like to be informed about normal and abnormal test results; and what further tests they would want to have in case of a normal FIT result.

### Numeracy skills

Numeracy was assessed using the item: ‘Which of the following numbers represents the biggest risk of getting a disease? 1 in 100, 1 in 1000, or 1 in 10’.^21^ Participants were scored as either correct or incorrect.

### Demographics and previous symptoms

Information was collected about the participants’ sociodemographic characteristics such as participant’s age, ethnic group (‘white British’ or ‘other white’, versus ‘black’ or ‘Asian’ or ‘mixed’ and ‘other’), marital status (‘married’ or ‘cohabiting’, versus ‘single’ or ‘divorced’ or ‘separated’ or ‘widowed’), having obtained at least A-levels (‘yes’ versus ‘no’), being in paid employment (‘yes’ versus ‘no’), and self-perceived risk of developing CRC. The latter was measured on a 5-point Likert scale ranging from ‘much lower than average’, ‘a little lower than average’, ‘about the same’, ‘a little higher than average’, to ‘much higher than average’. Participants were also asked to indicate how frequently they had experienced, in real life, common CRC-related symptoms such as constipation, diarrhoea, abdominal pain, bowel incontinence, blood in stool, and indigestion in the last 3 months: ‘not at all’, ‘occasionally’, or ‘frequently’.

### Statistical analysis

All statistical analysis was conducted with Stata/SE (version 15.1). The main outcome variable was the number of CRCs missed by FIT at which participants preferred CC over FIT. An adjusted ordered logistic regression was used to identify the potential factors associated with an individual's minimal acceptable sensitivity of FIT. A separate logistic regression analysed factors explaining not wanting to have CC or FIT if both miss 1% of CRCs. Owing to low frequencies, responses to the self-perceived risk questions were reclassified into three groups: (1) *'*much lower than average*’* combined with *‘*a little lower than average*’;* (2) ‘about the same’; and *(*3*) ‘*a little higher than average’ combined with ‘much higher than average’. Similarly, the answers to the CRC-related symptoms were reclassified into two groups, (1) ‘not at all’ versus (2) ‘occasionally’ and ‘frequently’. For both multivariate analyses, adjusted odds ratios (ORs) and 95% confidence intervals (CIs) were reported and a significance level of *P*<0.05 was used.

## Results

### Population characteristics

A total of 1837 responders were eligible to start the survey. Of those, 220 (11.98% of the total sample) dropped out. Of these 220 responders, 84 (38.2%) dropped out even before reading the vignette description, as shown in Figure 1. It can also be seen that 107/220 (48.6%) responders dropped out while reading the vignette and answering the associated comprehension question (see Supplementary file). A final 29/220 (13.2%) responders dropped out while answering the second comprehension question about the alarm symptom. In total, 1617 responders successfully finished the survey, of which 560 (34.6%) had at least one of the alarm symptoms, leaving a sample of 1057 responders for the final analysis. Table 1 gives an overview of the participants’ characteristics.

**Table 1.** Study sample‘s sociodemographic variables and symptoms in the last three weeks (*n* = 1057)

		Aged 40–49 years, *n* (%)	Aged 0–59 years, *n* (%)	Total, *n* (%)	*P* value
**Sex**					
	Men	281 (54.5)	283 (52.3)	564 (53.4)	0.484
	Women	235 (45.5)	258 (47.7)	493 (46.6)	
**Marital status**					
	Single or divorced or separated	166 (32.2)	196 (36.2)	362 (34.2)	0.165
	Married or cohabiting	350 (67.8)	345 (63.8)	695 (65.8)	
**Education**					
	No A-levels	170 (32.9)	225 (41.6)	395 (37.4)	0.004
	A-levels	346 (67.1)	316 (58.4)	662 (62.6)	
**Paid employment**					
	No	109 (21.1)	201 (37.2)	310 (29.3)	<0.001
	Yes	407 (78.9)	340 (62.8)	747 (70.7)	
**Ethnic group**					
	White	461 (89.3)	510 (94.3)	971 (91.9)	0.003
	BAME	55 (10.7)	31 (5.7)	86 (8.1)	
**Numeracy question**					
	Wrong	227 (44.0)	241 (44.5)	468 (44.3)	0.856
	Correct	289 (56.0)	300 (55.5)	589 (55.7)	
**Own perceived risk**					
	Lower	87 (16.9)	91 (16.8)	178 (16.8)	0.436
	Same	369 (71.5)	400 (73.9)	769 (72.8)	
	Higher	60 (11.6)	50 (9.2)	110 (10.4)	
**Constipation**					
	Not at all	429 (83.1)	424 (78.7)	853 (80.9)	0.065
	Occ/frequ	87 (16.9)	115 (21.3)	202 (19.1)	
**Diarrhoea**					
	Not at all	411 (80.0)	430 (79.6)	841 (79.8)	0.893
	Occ/frequ	103 (20.0)	110 (20.4)	213 (20.2)	
**Bowel incontinence**					
	Not at all	500 (97.3)	521 (96.5)	1021 (96.9)	0.459
	Occ/frequ	14 (2.7)	19 (3.5)	33 (3.1)	
**Indigestion**					
	Not at all	533 (79.8)	491 (73.9)	1024 (76.8)	0.024
	Occ/frequ	245 (20.2)	270 (26.1)	515 (23.2)	

BAME = black and minority ethnic. Occ/frequ = occassionally or frequently

### Preferences for FIT and CC

#### Preference for neither of the diagnostic tests

A total of 150 (14.2%) stated they would not want either test. Reasons for not wanting either test included perceiving both tests as insufficiently accurate (*n* = 61, 40.7%); being too scared of a bowel cancer diagnosis (*n* = 41, 27.3%); being afraid of test risks (*n* = 37, 24.3%); not needing the tests (*n* = 7, 4.7%); perceiving the tests as unpleasant, uncomfortable, and embarrassing (*n* = 5, 3.3%); or ‘other’ reasons (*n* = 14, 9.3%).

A total of 907 participants (85.8%) indicated either a preference for CC or FIT when both missed 1% of CRCs. The logistic model in Table 2 shows that the likelihood of not wanting either test was positively associated with having a black or Asian minority ethnic (BAME) background (OR 3.49; 95% confidence intervals [CI] = 2.07 to 5.89), having lower numeracy skills (OR 2.54; 95% CI = 1.74 to 3.71), and having a higher self-perceived CRC risk (OR 2.35; 95% CI = 1.20 to 4.62).

**Table 2.** Multivariate models explaining not wanting to have CC or FIT and switching point

	Logistic model: not wanting any test	Ordinal model: switching point
	**Odds ratio**	**95%** **CI**	**Odds ratio**	**95%** **CI**
**Sex**				
Male	Ref		Ref	
Female	0.813	0.555 to 1.192	1.413	1.109 to 1.800^a^
**Age, years**				
40–49	Ref		Ref	
50–59	0.821	0.564 to 1.194	1.008	0.792 to 1.282
**Education**				
No A-levels	Ref		Ref	
A-levels	0.864	0.586 to 1.274	1.222	0.950 to 1.572
**Paid employment**				
Yes	Ref		Ref	
No	1.294	0.865 to 1.938	1.208	0.925 to 1.579
**Marital status**				
Single or divorced or separated	Ref		Ref	
Married or cohabiting	0.937	0.640 to 1.373	0.807	0.629 to 1.035
**Ethnic group**				
White	Ref		Ref	
BAME	3.492	2.071 to 5.888^a^	0.676	0.402 to 1.134
**Numeracy question**				
Correct	Ref		Ref	
Wrong	2.539	1.737 to 3.712^a^	0.736	0.576 to 0.940^b^
**Own perceived risk**				
Lower	Ref		Ref	
Same	1.178	0.700 to 1.981	0.983	0.719 to 1.343
Higher	2.352	1.199 to 4.617^b^	0.546	0.336 to 0.887^b^
**Constipation**				
Not at all	Ref		Ref	
Occasionally	1.056	0.637 to 1.751	0.848	0.614 to 1.169
**Diarrhoea**				
Not at all	Ref		Ref	
Occasionally	0.856	0.514 to 1.424	0.939	0.691 to 1.277
**Bowel incontinence**				
Not at all	Ref		Ref	
Occasionally	0.396	0.087 to 1.810	0.588	0.296 to 1.166
**Indigestion**				
Not at all	Ref		Ref	
Occasionally	0.769	0.476 to 1.242	1.572	1.178 to 2.097^a^
	1048		903	

^a^ *P*<0.01. ^b^ *P*<0.05.

BAME = black and minority ethnic. CI = confidence intervals. Ref = reference.

#### Alternative actions

Participants reported that they would go back to their GP (*n* = 80, 53.3%), monitor their symptom(s) (*n* = 53, 35.3%), try to manage the symptoms on their own (*n* = 26, 17.3%), do nothing (*n* = 22, 14.7%), avoid thinking about their symptom(s) (*n* = 15, 10%), or take ‘another’ action (*n* = 3, 2.0%) instead of having either an FIT or CC.

#### Reasons for preferring CC

The main reasons for choosing CC over FIT when both tests miss 1% of CRCs were: perceiving CC to be more thorough (*n* = 98, 59.0%), better at finding polyps (*n* = 62, 37.4%), or more able to remove polyps (*n* = 49, 29.5%); to avoid uncertainty associated with an abnormal FIT result (*n* = 34, 20.5%); being more familiar with CC (*n* = 34, 20.5%); not wanting to handle stool (*n* = 19, 11.5%); lack of trust in FIT (*n* = 14, 8.4%); or ‘other’ reasons (*n* = 7, 4.2%).

#### Preference for FIT

**Figure 3.**
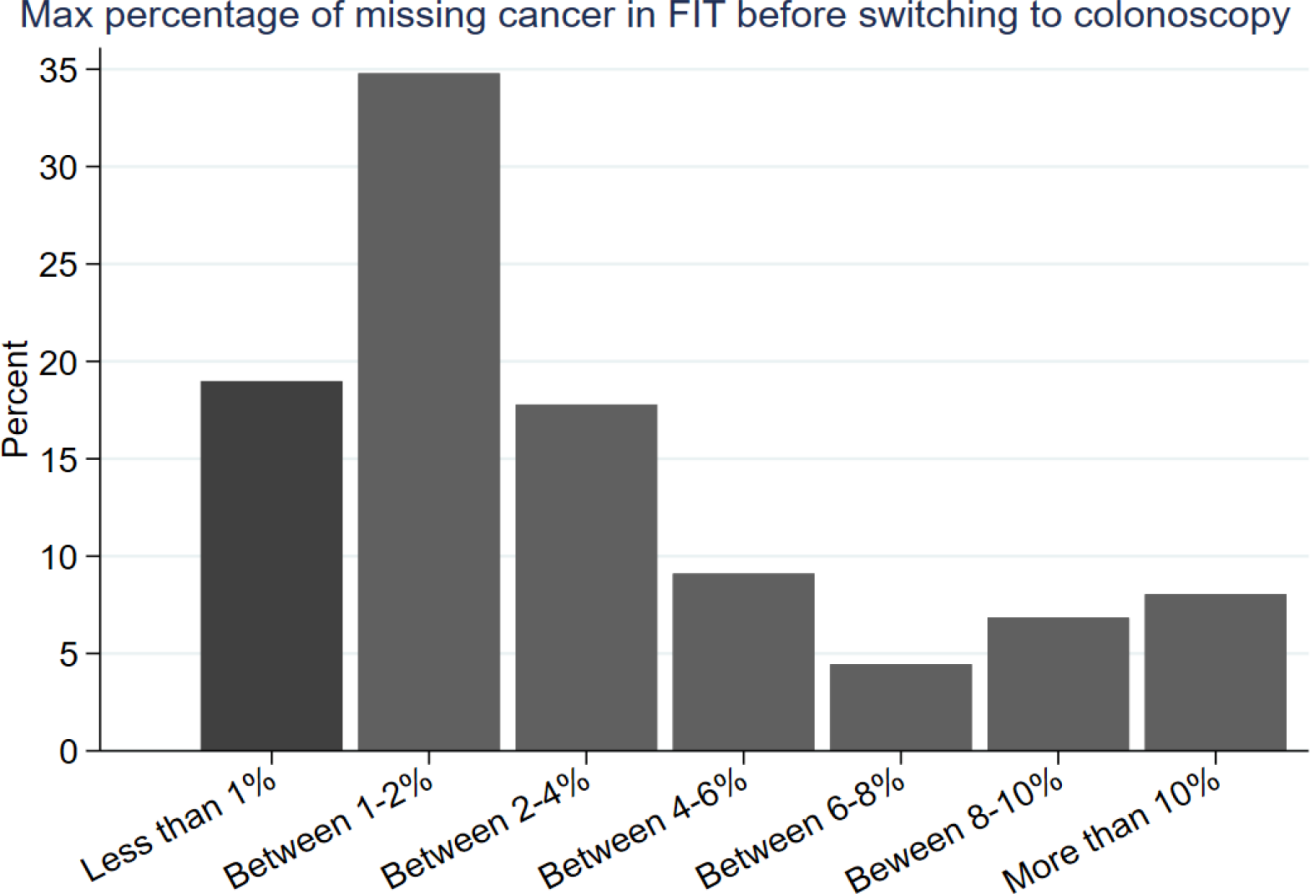
Switching point for responders’ faecal immunochemical test (FIT) preference over colonoscopy (CC) (*n* = 1057). Those who preferred FIT over CC in choice one were asked to choose between a CC that misses 1% of colorectal cancers (CRCs) and an alternative FIT that misses 2% of CRCs in choice 2. Participants who kept choosing FIT over CC were presented with new scenarios in which the number of CRCs missed by FIT increased constantly by 2%. In the final scenario, FIT was reported to miss 10% of CRCs. The method let the authors infer the maximum acceptable number of CRCs missed by FIT.

Figure 3 shows at what threshold of missed CRCs participants preferred CC over FIT. The majority (*n* = 741, 70.1%) preferred FIT over CC when both tests missed 1% of CRCs. This reduced to 427 (40.4%) when FIT missed 2% of CRCs. Only 7.1% (*n* = 75) preferred FIT when it was described to miss 10% of CRCs. Tolerating a higher level of CRCs missed by FIT was associated with being female (OR 1.41; 95% CI = 1.11 to 1.80) and suffering occasionally or frequently from indigestion (OR 1.57; 95% CI = 1.18 to 2.10). Conversely, having lower numeracy skills (OR 0.74; 95% CI = 0.58 to 0.94) and having a higher self-perceived CRC risk (OR 0.55; 95% CI = 0.34 to 0.89) were associated with switching sooner to CC.

#### Preferences of communicating FIT result

The majority of participants who selected FIT at least once in the choice task (*n* = 461, 62.2%) would prefer to send it to a laboratory using freepost instead of returning it to their local GP practice in person (*n* = 280, 37.9%). If the FIT result was normal, most participants would prefer to be informed by letter (*n* = 312, 42.1%) or online (*n* = 226, 30.5%). Conversely, if the test result was abnormal, most participants would prefer to be informed face to face (*n* = 241, 32.5%) rather than by a letter (*n* =2 09, 28.2%) or over the phone (*n* = 167, 22.6%). Additionally, the majority would like to discuss the abnormal result with a specialist (*n* = 410, 55.3%) instead of their GP (*n* = 326, 44.0%).

## Discussion

### Summary

This study showed that while most responders would prefer FIT over CC if both tests miss 1% of CRCs, a large proportion of participants switched their preference to CC as soon as FIT missed more CRCs. This was particularly the case for participants who reported lower numeracy skills and among those who perceived their risk of CRC to be higher. Females and those occasionally or frequently suffering from indigestion were more likely to accept a larger number of false negative FIT tests. A smaller but important subgroup of people exhibited a strong preference for non-invasive testing (that is, using FIT over CC) even if the test missed 10% of CRCs. Out of the 75 who always chose FIT, most were female (54.7%), married or cohabiting (61.3%), white (92.0%), had at least A-levels (68.0%), were in paid employment (60.0%), and got the numeracy question right (60.0%).

Conversely, it was concerning that 150 participants (14.2%) reported that they would not undergo either of the tests to rule out CRC. The reasons for this were diverse and, in some cases, counterintuitive, showing the need to explore reasons for declining investigate testing, especially as many of the groups (for example, those with low literacy, and those from a minority ethnic background) who were overrepresented have low engagement rates with cancer screening programmes.^22^

### Comparison with existing literature

Many of the findings are in line with a previous study showing that most patients would be willing to undergo a CC when they perceived their risk of CRC to be more than 1%.^18^ They also indicate that prospective patients — like GPs,^12^ and people invited for CRC screening^15–17^ — have concerns about the ability of FIT to detect CRC accurately. Furthermore, as reported in studies looking at uptake of screening tests for CRC, fear of the result was one of the most commonly reported reasons by responders who did not want to have either CC or FIT.^23^ There are also strong parallels with studies looking at preferences for CC versus FIT in the symptomatic context.^24,25^ Furthermore, there is the observation that women had a higher tolerance for missed cancers before switching to CC. This finding was in line with previous research demonstrating that women report more pain during CC^26^ and have a stronger preference for non-invasive CRC diagnostic modalities.^27^ Similarly, it was not surprising that responders with higher perceived CRC risk were more likely to switch sooner to CC given previous evidence showing that high-risk patients are more likely to prefer CC compared with FIT.^24^ In contrast, the finding that responders with low numeracy switched sooner to CC was novel and requires further investigation.

With regards to numeracy, it was important to note that only 55% of responders answered the numeracy question correctly; this was in line with a previous study using a comparable sample of UK adults in a similar age range,^28^ which may have to do with specific characteristics of the sample population. However, it also demonstrates the need to review specifically the way that information about risk (often presented in numerical form) is communicated to the public.

### Strengths and limitations

This was the first study that aimed to elicit public preferences for FIT versus CC in the symptomatic context and to determine the point at which patients would prefer a more invasive procedure to avoid potentially missing CRC. These findings are important because of the urgent need to reduce the diagnostic burden on CC by using less resource-intensive modalities such as FIT. Furthermore, the investigation of preferences for the process of delivering FIT is timely given the current implementation of this test in primary care. Here the preference for accuracy level could be reflected directly with the choice of referral threshold for FIT. The findings indicate that, to meet patient preferences, the referral threshold of FIT should be low to reassure patients that the test will be no more likely than CC to miss CRC. The information about demographic antecedents of these preferences should help local authorities and commissioners provide services that are tailored to their specific population.

The major limitation of this study was that choices were focused to understand preferences for testing in relation to missed cancers, or to the sensitivity of FIT versus CC. The next step would be to conduct a discrete choice experiment in which several test attributes were manipulated, including test specificity. Previous studies have used this design to identify preferences for different CRC screening modalities and service specification within a screening modality.^29^

Another limitation was that this study focused on intention, which may not translate into real behaviour (that is, people who indicated that they prefer a FIT that misses 10% of CRCs over a CC that only misses 1% may choose differently if they find themselves in the real situation).^30^ Relatedly, the scenario setting was such that only FIT efficacy was variable, while the assumption was that CC is highly effective; it is known, however, that CC could miss up to 10% of cancers,^31^ so the follow-up survey should investigate whether the outcomes are similar when CC efficacy varies while FIT remains the same. Finally, it is important to note that responders who had recently experienced alarm symptoms were excluded. One may argue that these are the very people this study was aimed at; it was important, however, to ensure that responders were not influenced by their own experiences, but that every responder would make a choice based on the symptoms presented to them in the vignette. However, future research could be selectively sample responders with recent alarm symptoms to examine their preferences based on their recent symptoms and experiences.

### Implications for practice

This study highlights important insights into people’s preferences of how to return their test kit, and how to receive and follow-up its results, which is in line with preferences expressed in CRC screening programmes. In the English CRC screening programme, people are asked to return the test kit in the mail and receive their results as part of a letter. People who receive an abnormal test result are then invited to attend an appointment with a screening practitioner to discuss follow-up testing. It was notable that in the surveyed cohort with imagined symptoms, most responders would prefer to return their kit by mail and receive a normal test return by letter but want to be informed about an abnormal test result face to face by a specialist rather than their GP. It should also be noted, however, that there was no universal preference or clearly dominating options, meaning that a one-size-fits-all approach might disenfranchise a subsection of the population. Local commissioners should, therefore, be encouraged to tailor implementation and service delivery to their local demographic.

Perhaps most interesting is the observation that participants’ ‘switching point’, or the tolerance for false negatives, was associated with being female and a previous history of indigestion. It is particularly interesting that the combination was specific to indigestion rather than any other symptom. The role of other comorbidities could be an important moderator. Previous studies have indicated that comorbidities can act as a prompt or barrier to help-seeking.^32^ In the case of indigestion, people may already have had previous investigation and, therefore, have a stronger preference for a non-invasive testing modality.

This study found that, as long as FIT can offer the same level of reassurance as CC in identifying patients who are highly unlikely to have CRC, it will be highly acceptable and generally preferred. It was also found, however, that the majority of people switched their preference back to the gold standard test as soon as FIT started missing even one additional cancer; thus, for the implementation of FIT to be successful, it will be crucial that FIT has a high accuracy level and that implementation plans will take into account local population preferences.
